# Supplementary material for: Content-rich biological network constructed by mining PubMed abstracts
Source: BMC Bioinformatics. 2004 Oct 8;5:147. doi: 10.1186/1471-2105-5-147 (PMC528731; doi:10.1186/1471-2105-5-147)
Supplement: Additional File 2 — The original results of the above study (non-essential files are deleted to keep the file size under the limit set by BMC bioinformatics). [file 1471-2105-5-147-S2.bz2 › chilibotAdditionalFile2/dip10/432ID10910894E10362/html/CCR8_CCL17.html]

 


 **CCR8** and **CCL17** 
  
Found 21 abstracts in PubMed, retrieved 10.  
 

 What does Google say? 
 PDF only 
| .edu only 

---

**Interactive relationship** (e.g. stimulation, inhibition, etc)

**Neutral relationship**- ... interactions of CCR4 with MDC CCL22 or TARC  [ **CCL17** ]   **CCL17** ,  **CCR8**  with I 309 CCL1, and CRTH2 with prostaglandin D 2 play a critical role in the allergen induced recruitment of Th2 cells in the target tissues of allergic inflammation.  Ref: 12009564 Mol Immunol, 2002
- TARC  [ **CCL17** ]  interacts primarily with the CCR4 receptor and to a lesser extent with the  **CCR8**  receptor.  Ref: 12499558 Acta Crystallogr D Biol Crystallogr, 2003
- TARC  [ **CCL17** ]  interacts primarily with the CCR4 receptor and to a lesser extent with the  **CCR8**  receptor.  Ref: 12832759 Acta Crystallogr D Biol Crystallogr, 2003
- Thymus and activation regulated chemokine  [ **CCL17** ]  TARC  [ **CCL17** ]   **CCL17**  is a member of the CC chemokines and is a chemoattractant for CC chemokine receptor 4 CCR4 and CC chemokine receptor 8  [ **CCR8** ]   **CCR8**  expressing cells.  Ref: 12522366 J Am Acad Dermatol, 2003
- Treg cells specifically express the chemokine receptors CCR4 and  **CCR8**  and represent a major subset of circulating CD4 T cells responding to the chemokines macrophage derived chemokine MDC CCL22, thymus and activation regulated chemokine  [ **CCL17** ]  TARC  [ **CCL17** ]   **CCL17** , I 309 CCL1, and to the virokine vMIP I ligands of CCR4 and  **CCR8** .  Ref: 11560999 J Exp Med, 2001
- T regulatory cells, which inhibit dendritic cell function and are probably involved in the termination of ACD, are sensitive to MCP 1, MIPs and TARC  [ **CCL17** ] , but express high levels of  **CCR8**  and are more specifically attracted by I 309.  Ref: 11876523 Arch Dermatol Res, 2002
